# Programmed cell death throughout life influences the longevity of a defective mitochondrial mutant in *C. elegans*

**DOI:** 10.17912/micropub.biology.001686

**Published:** 2025-08-04

**Authors:** Sumino Yanase, Rea Yamaguchi, Kayo Yasuda, Naoaki Ishii

**Affiliations:** 1 Daito Bunka University, School of Sports & Health Science, Iwadono 560, Higashi-matsuyama, Saitama 355-8501, Japan; 2 Tokai University, School of Health Studies, Kita-kaname 4-1-1, Hiratsuka, Kanagawa 259-1292, Japan

## Abstract

In *C. elegans* , the *mev-1* gene mutation leads to increased mitochondrial dysfunction and embryonic abnormal apoptosis, thereby shortening the lifespan. A mutation in the *ced-3* gene encoding an ortholog of mammalian caspases reduces the excessive embryonic apoptosis and recovers the lifespan of the *mev-1* mutant. Here, we report the difference between temporary in early development and continuous knockdowns of the *ced-3* gene. We found that CED-3 /caspase is essential to the abnormal apoptosis in the *mev-1* mutant, not only during development but also during aging. These findings indicate an association of CED-3 /caspase with age-related cellular dysfunction even in somatic cells.

**Figure 1. Difference in lifespan of the mev-1 mutant treated with embryonic RNAi by soaking and continuous feeding RNAi of the ced-3 gene.**
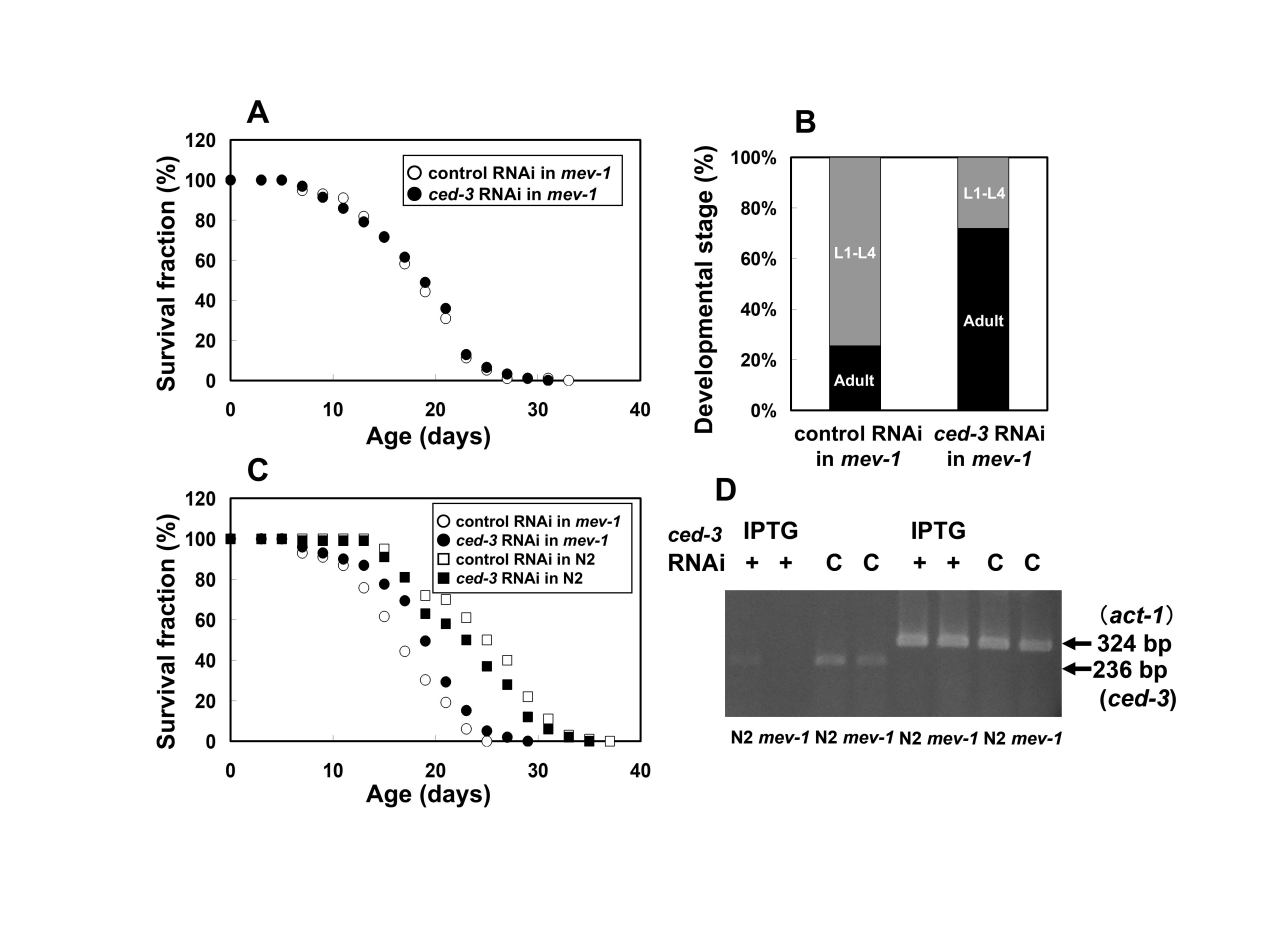
**A)** Embryonic RNA interference (RNAi) by soaking of the *ced-3* gene in the *mev-1* mutant had no effect on longevity at 20°C. Mean lifespans ± standard deviation (S.D.) were 18.9 ± 5.4 days in the *mev-1* mutant treated with soaking RNAi and 18.7 ± 5.2 days in the control worms, respectively. In the survival curves, the lifespans of the wild-type N2 have been omitted because there was no difference between those with and without RNAi treatments. **B)** However, larval development after 72 hours from the embryonic stage at 20°C in the *mev-1* mutant treated with soaking RNAi recovered to levels close to 100% of those in the wild-type. **C)** In contrast, continuous feeding RNAi of the *ced-3* gene extended the lifespan of the *mev-1* mutant compared with embryonic RNAi by soaking. Mean lifespans ± S.D. were 19.3 ± 4.9 days in the *mev-1* mutant treated with feeding RNAi and 17.1 ± 4.8 days in the control worms, respectively. In the wild-type N2, mean lifespans ± S.D. were 23.5 ± 5.7 days treated with feeding RNAi and 25.1 ± 5.8 days in the control worms, respectively.. Comparison of the means by Student’s *t* -test revealed a significant difference compared with the control for the *mev-1* mutant treated with feeding RNAi ( *P* < 0.005). **D)** Amplified DNA fragments of the reverse transcription polymerase chain reaction (RT-PCR) analysis were detected using an agarose gel electrophoresis. Feeding RNAi of the *ced-3* gene revealed knockdown of the target gene compared with mRNA expression levels of *act-1* gene as a positive control.

## Description

The *mev-1* mutant in the nematode *Caenorhabditis elegans* ( *C. elegans* ), which was isolated as a methyl viologen (paraquat)-sensitive mutant, is hypersensitive to oxygen and has decreased longevity compared with the wild-type N2 (Ishii et al., 1990). The gene encodes a large subunit of the enzyme succinate dehydrogenase cytochrome *b* , which is a component of complex II in the mitochondrial electron transport chain (Ishii et al., 1998). In the *mev-1* mutant, mitochondrial superoxide production levels are increased compared with the wild-type and also show an increasing dependence on hyperoxia (Senoo-Matsuda et al., 2001; Yasuda et al., 2011). However, the expression levels of antioxidant genes encoding superoxide dismutases and catalases as well as their activities were similar to those in N2 (Yanase et al., 2002; Yanase and Ishii 2008). Moreover, excessive embryonic apoptosis in the *mev-1* mutant depends on the *ced-3* gene, which is a homolog of mammalian caspases 3/6/7 and an integral element of the programmed cell death (PCD) pathway including the *ced-9 / ced-4 / ced-3* genes in *C. elegans* (Ellis and Horvitz, 1986; Senoo-Matsuda et al., 2003). The genetic suppression of the *ced-3* gene partially restores the lifespan of the *mev-1* mutant to that of the wild-type. Previous reports suggested that the delayed embryonic development is due to CED-3-dependent abnormal apoptosis in the *mev-1* mutant, and plays at least a partial role in the short lifespan (Senoo-Matsuda et al., 2003). Here, we examined whether embryonic RNA interference (RNAi) of the *ced-3* gene rescues not only the developmental delay but also the short lifespan of the *mev-1* mutant. To treat the embryonic RNAi, we first used the soaking RNAi of the *ced-3* gene with larval stage 4 (L4) worms in the *mev-1* mutant and measured the lifespan of the first filial (F1) worms. After hatching, the larvae in the *mev-1* mutant grow at a slightly slower rate compared with the wild-type. That is, larval development until the adult stage in the *mev-1* mutant is delayed by roughly half to a full day, whereas most wild-type worms attained adulthood after 3 days at 20°C (Ishii et al., 1990; Yasuda et al., 2011). After 72 hour, the developmental stage of wild-type N2 was 100% adult (Baruah et al., 2014). Despite restoring the developmental delay in the *mev-1* mutant by using embryonic RNAi by soaking of the *ced-3* gene, there was no difference in lifespans between those with and without the RNAi treatment ( **Figure 1A and B** ) (Yasuda et al., 2011; Baruah et al., 2014). In contrast, using bacterial feeding RNAi to continuously knock down the *ced-3* gene in the *mev-1* mutant extended the lifespan significantly compared with the RNAi control ( **Figure 1C** ) dependent on suppressing the expression of the *ced-3* gene ( **Figure 1D** ). Thus, we found that inactivation of the *ced-3* gene not only during embryonic developmental stage but also throughout life is required to extend the lifespan of the *mev-1* mutant. Taken together, these findings suggest that CED-3 /caspase in PCD pathway is not only essential for abnormal developmental apoptosis in the embryonic and larval stages but also crucial to shortening the lifespan of the *mev-1* mutant through mitochondrial dysfunction, even after the development to the adult stage. Therefore, we revealed that the *ced-3* -dependent apoptosis plays at least a partial role in aging and longevity, even in somatic cells of *C. elegans* .

## Methods

**Nematode culture and media**

The wild-type N2 and *mev-1 (kn1)* strains were grown at 20°C on nematode growth medium (NGM) agar plates with *Escherichia coli* ( *E. coli* ) OP50 strain, which is a uracil auxotroph that inhibits growth on NGM plates until RNAi treatment is initiated (Lewis and Fleming 1995).

**Soaking and feeding RNA interference**

Soaking RNAi was performed to treat 10 L4-stage *mev-1* mutant hermaphrodites for 24 hours at 20°C with double-stranded RNA (dsRNA) dissolved in 4 µL of soaking buffer (10.9 mM Na _2_ HPO _4_ , 5.5 mM KH _2_ PO _4_ , 2.1 mM NaCl, 4.7 mM NH _4_ Cl, 3 mM spermidine, and 0.05% gelatine) containing 0.6 µL of DOTAP Liposomal Transfection Reagent (Roche Diagnostics GmbH, Mannheim, Germany) (Fire et al., 1998; Maeda et al., 2001). Each strand of dsRNA was synthesized using in vitro transcription (IVT) with either T3 or T7 RNA polymerase from a template DNA derived from wild-type N2 . Either ced-3 G.T7.F-primer or ced-3 G.T3.R-primer was used for each IVT. As a control, *mev-1* worms treated similarly in soaking buffer (containing DOTAP) without dsRNA. After soaking RNAi, the hermaphrodites were moved to new plates and laid eggs. These F1 offspring produced after 24 hours of egg laying by the hermaphrodites were utilized to measure the developmental stage and lifespan (Maeda et al., 2001).

To prepare feeding RNAi plates, bacterial clone in *E. coli* HT115 (DE3) strain was obtained from the *C. elegans* RNAi feeding library made by Arlinger's groups at the Gurdon Institute, University of Cambridge through K.K. DNAFORM (Yokohama, Japan) and verified by one-pass sequencing (Kamath et al., 2003). The bacterial clone was seeded on NGM plates containing 100 µg/mL ampicillin with isopropyl-β-D-thiogalactopyranoside (IPTG; Wako Pure Chemical Industries Ltd., Osaka, Japan). As a control, bacterial plates without IPTG were used similarly for feeding RNAi. For feeding RNAi, eggs of gravid hermaphrodites were harvested using an alkaline sodium hypochlorite washing, before which age-synchronous culture of first-stage larvae (L1) was performed in S-buffer (Sulston and Brenner, 1974; Lewis and Fleming 1995). The synchronized L1-stage larvae were transferred to the feeding RNAi plates and cultured at 20°C until the young adult stage. These young adult stage hermaphrodites were utilized to measure the mRNA expression levels of the *ced-3* gene as well as the lifespan in fresh feeding RNAi plates.

**Lifespan assay**

After the F1 offspring from the *mev-1* mutant treated with soaking RNAi reached the L4 stage, 10 larvae per plate were transferred to 10 φ35-mm NGM agar plates. After the hermaphrodites reached the young adult stage in the feeding RNAi plates, 10 worms per plate were transferred to φ35-mm plates with the bacterial clone. The lifespan of the hermaphrodites at 20°C was measured using a total of 100 worms per trial (Ishii et al., 1990). To prevent progeny production, 40 µM 5-fluoro-2'-deoxyuridine (FUdR; Wako Pure Chemical Industries Ltd., Osaka, Japan) was added to the plate after worms reached adult stage (Hosono, 1978). We confirmed that the FUdR treatment did not impact RNAi by the bacterial clone. The lifespan measurements were performed at least three times.

**Developmental assay**

The percentage of worms at each developmental stage of larva or adult was quantified for the *mev-1* mutant after embryonic RNAi by soaking of the *ced-3* gene. After soaking RNAi treatment, the F1 hermaphrodites laid eggs on the NGM agar plates. We separated the eggs on the NGM agar plates each day and determined the developmental stage of about 50 worms after 72 hours at 20°C on the NGM plate, using a microscope (Yasuda et al., 2011; Baruah et al., 2014). The developmental measurements were performed at least three times.

**Total RNA isolation and quantitative RT-PCR**

Total RNA of the worms treated with feeding RNAi was prepared using a PureLink RNA Mini-kit (Ambion Inc., Austin, TX, USA). cDNA was synthesized by reverse transcription (RT) from them as previously reported (Yanase and Ishii, 2008). The cDNA was employed as a template for subsequent polymerase chain reaction (PCR) to measure the expression of *ced-3* and *act-1* genes as a positive control.

## Reagents

**The strains and genotypes**

The wild-type N2 strain of *C. elegans* var. Bristol and TK22 *mev-1 ( kn1 )* mutant were acquired from the Caenorhabditis Genetic Center (CGC; University of Minnesota, Minneapolis, MN, USA).

**Sequences of primers for RNA polymerization and PCR**

ced-3 G.T7.F-primer: TAATACGACTCACTATAGGGGCAGCAAGTGTGGAGAAAG

ced-3 G.T3.R-primer: ATTAACCCTCACTAAAGGGACAAGTACCTCTGGCATCTG

ced-3 .n1286.F-primer: CAAGTGTGGAGAAAGAAGCC

ced-3 .n1286.R-primer: GGCATCTGTTTCAAAATATTCG

act-1-1.F-primer: CCACGTCATCAAGGAGTCAT

act-1-2.R-primer: GGAAGCGTAGAGGGAGAGGA
